# Emergency medicine in Nepal: are we going the right way and fast enough?

**DOI:** 10.1186/s12245-023-00553-6

**Published:** 2023-11-02

**Authors:** Sanjeeb S. Bhandari, Subarna Adhikari

**Affiliations:** 1 Collaboration for Emergency Care Nepal, Pokhara, Nepal; 2 Collaboration for Emergency Care Nepal, Kathmandu, Nepal

**Keywords:** Emergency medicine residency, Trauma, Natural disasters, Pandemics

## Abstract

Nepal is a landlocked country with variable topography including the world’s tallest mountains, tropical forests, and Gangetic plains. This topography leads to diverse healthcare needs, from tropical diseases in plains, to road traffic accidents in hills, and to disaster-related emergencies from floods and landslides during monsoon season and unpredictable earthquakes. Emergency medicine care is underdeveloped and is currently being provided by general practitioners. The capital city Kathmandu has a few tertiary healthcare centers with emergency medicine fellowship-trained general practitioners staffing them, but most of the hospitals outside of Kathmandu are staffed by minimally trained or untrained medical officers. Even though emergency medicine is recognized as a specialty, Nepal still does not have an emergency medicine residency training program. To improve emergency care in the country, the government of Nepal should be focused on facilitating the start of emergency medicine residency in Nepal.

**Graphical Abstract:**

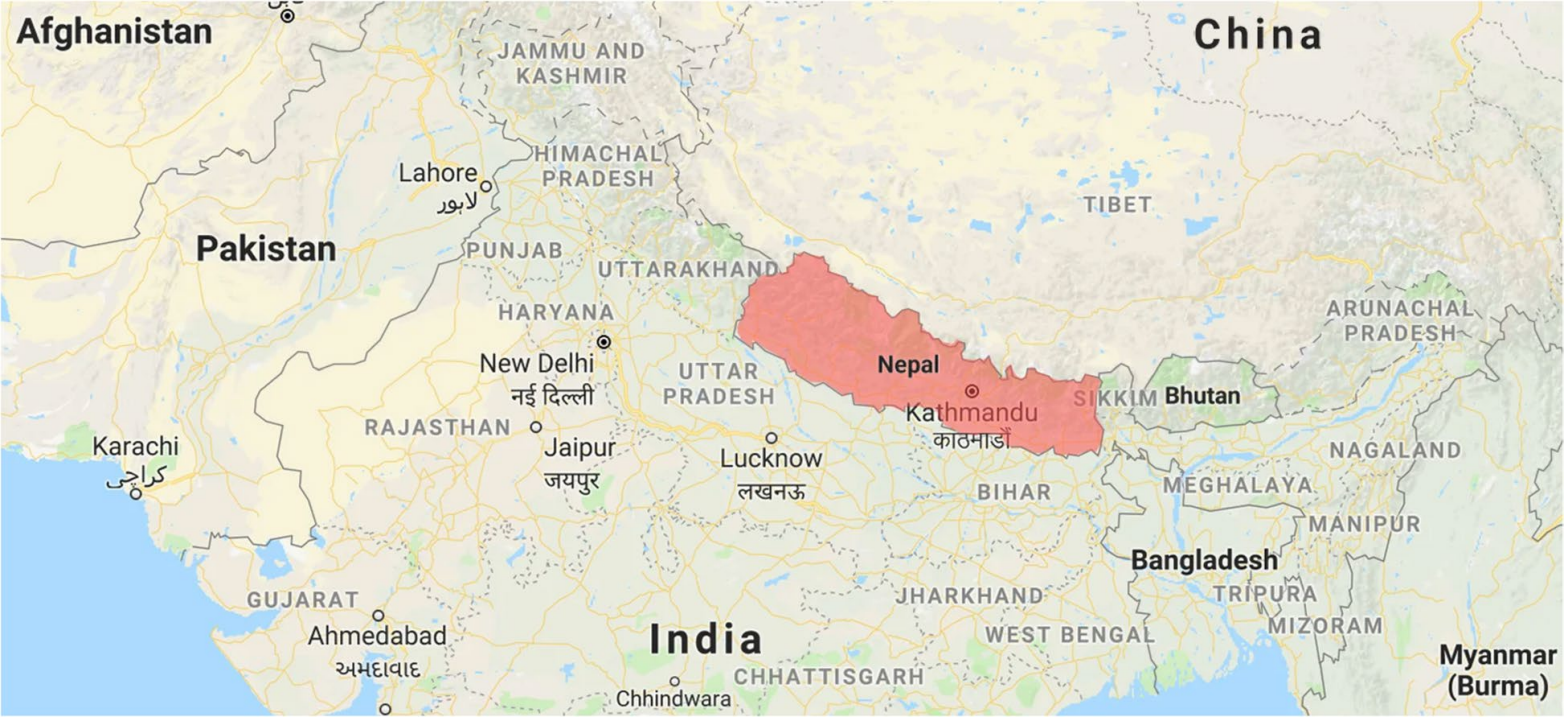

## Background

Nepal is a landlocked country between India and China. The topography is extremely variable, with the northern Himalayas, middle Mahabharata hills, and southern Gangetic plains. This diverse topography contributes to a wide range of health problems ranging from malaria, dengue, and other tropical diseases in the southern plains, to malnutrition in the mountains, and to road traffic accidents due to difficult terrain in the middle hilly region. Nepal also lies on the fault line between the Asian and Indian tectonic plates making it one of the most vulnerable regions to earthquakes. Mountains also act as a barrier for rising moisture from the Bay of Bengal as a result of which Nepal gets large amounts of monsoon rain in summer. Coupled with steep terrain, this makes it more vulnerable to other natural disasters like landslides in the middle hilly region and floods in the southern plains. The population of Nepal in 2019 was 28.61 million with 21.45% of the population living in the urban areas, according to the World Bank report.

### Healthcare delivery system

The healthcare system in Nepal is regulated at three different levels according to the constitution of Nepal 2015. The municipal hospitals, primary healthcare centers (PHCs), health posts, and other hospitals with less than 25-bed capacity are approved and regulated by local-level government authorities. Private hospitals with less than 200 beds are regulated by the provincial government. The Ministry of Health of the government of Nepal regulates all tertiary level hospitals, government-run hospitals, private hospitals, and all teaching hospitals. Nepal army runs army hospitals where people who have served in the army and their families get highly subsidized service. They have also started serving the public recently. Nepal police and the armed police force also have their own hospitals, serving their service members and their families at highly subsidized rates. The majority of the population is uninsured despite the fact that recent governments have launched government-run health insurance, which has been helpful to the population who have decided to enroll themselves in the program, but it is still inaccessible to the populace living in rural regions. Most of the healthcare cost comes out of pocket. Patients should pay upfront for diagnostic techniques and therapeutic procedures, oftentimes requiring patients to wait or stay hospitalized for days before they can arrange the cash payment.

### The emergency medical system

The Tribhuvan University Teaching Hospital (TUTH) started residency training in general practice in 1982 and has been providing emergency room physicians for the country. General practitioners (GPs) working in urban areas have been providing emergency room service, with medical officers (MO) working under them, as there was no formal emergency medicine residency in Nepal [1] until recently. Medical officers are medical school graduates with 1 year of internship training in all the medical and surgical departments. MOs serve as middle-level providers in different departments. However, most of the rural health centers are run by health assistants (HA) with only 2 years of high school level training or community medical assistants (CMA) with only 15–18 months of training. The primary health centers are run by MOs. The pharmacies, which also function as street corner clinics, are run by CMAs, HAs, or pharmacists which are often the first place most of the population seeks preliminary care. Neurology specialty hospitals deal with neurological emergencies, and cardiology centers deal with cardiological emergencies. There is only one affordable pediatric hospital, Kanti Children’s Hospital, the emergency department of which is also staffed by medical officers with pediatricians supervising them. Realizing the need for trauma care, the government of Nepal started the construction of the National Trauma Center in 1997, with financial aid from the government of India, which was completed in 2009 and only came into service in 2015, due to slow bureaucracy. The trauma care is headed by the Department of Orthopedics in the National Trauma Center.

Emergency medical services are still in the developing stage. Very few ambulance services have an emergency medicine technician (EMT) or a paramedic. Most of the ambulances are owned by a hospital, manned only by just a driver, with no emergency medical training. A nonprofit ambulance service called “Nepal Ambulance Service” runs with an EMT/paramedic in it [2]. It started in 2011 with 5 ambulances in Kathmandu Valley. It has since expanded to three other cities with one ambulance in each city. One study done in Patan Hospital showed that only 9.9% of the patients coming to the emergency department had arrived via any form of ambulance, whereas 53.6% reported arriving via taxi [3]. Helicopter-based emergency medical services exist via private helicopter companies, with no paramedic/medic in it. There are instances when a helicopter has a GP in them if contracted by the hospital where the patient is being transferred. It is also very expensive and is out of reach of the average population. It is mostly utilized by tourists with health insurance or by affluent people who can afford to get evacuated from the mountains or from smaller towns/cities to the bigger ones in case of emergency [4].

### Emergency medicine education and training programs

Nepal has a formal accreditation process for graduate medical education for different specialties. However, there is no standardized emergency medicine residency program. Long after TUTH started producing general practitioners in 1982, BP Koirala Institute of Health Sciences (BPKIHS) and the National Academy of Health Sciences (NAMS) started their own GP residency [5]. The governing body of doctors in Nepal, the Nepal Medical Council (NMC), recognized emergency medicine as a specialty in December of 2013, after application from two EM residency-trained physicians who returned after completion of specialty training in China [1]. TUTH started a fellowship level 3-year subspecialty training in emergency medicine for GP-trained doctors, graduating the first two doctors in 2015. BPKIHS and Patan Hospital started 18 months of emergency medicine fellowship training for general practitioners, graduating their first graduates in 2015 [1]. Chitwan Medical College is involved in a 4-year hybrid emergency medicine residency program for medical graduates where they get the 1st and 4th year of training in Chitwan Medical College Teaching Hospital and get a second and third year of training in Doncaster and Bassetlaw Hospital in England [6]. This program started in 2018 and is yet to graduate an EM physician. The new body is formed by the government of Nepal called the Medical Education Commission (MEC) to regulate medical education in Nepal and has recently granted permission for emergency medicine residency to TU Teaching Hospital which enrolled the first resident in May 2023.

There is no formal EMT or paramedic course in Nepal except for the sporadic EMT training conducted by the Nepal ambulance service for their ambulance staff.

## Conclusions

Emergency medicine is an established medical subspecialty in the developed parts of the world, and it forms the backbone of the health system along with emergency medical services. Even though it is one of the youngest medical specialties, it has been recognized as a specialty in the USA and the UK since the 1970s. Residency training started in those countries around the same time. Residency training in emergency medicine started in developed Asian countries in the 1980s. In India, a formal emergency medicine residency program started in 2011 [7].

Even though the post-residency training programs are producing some emergency medicine doctors in Nepal, these are inefficient in terms of time and resources and are at best, temporary solutions. It is high time Nepal envisioned a residency training program in emergency medicine. Time and again, disasters like earthquakes and pandemics have revealed shortcomings in the healthcare system of Nepal [8, 9]. The strengthening of healthcare should start with grassroot programs to train first responders, formal EMT training programs, community-level EMS, and initiation of a standardized emergency medicine residency, which should be based on an evidence-based learning environment and simulation-based hands-on practice.

## Data Availability

Not applicable.

## Abbreviations

BPKIHS: Bishweshwar Prasad Koirala Institute of Health Sciences
CMA: Community medical assistant
EMT: Emergency medicine technician
GP: General practitioner
HA: Health assistant
MEC: Medical Education Commission
MO: Medical officers
NAMS: National Academy of Medical Sciences
NMC: Nepal Medical Council
PHC: Primary healthcare center
TUTH: Tribhuvan University Teaching Hospital
